# Effect of *Lactobacillus fermentum* HFY03 on the Antifatigue and Antioxidation Ability of Running Exhausted Mice

**DOI:** 10.1155/2021/8013681

**Published:** 2021-09-28

**Authors:** Junxiao Zhang, Ling Chen, Lingyan Zhang, Qiuping Chen, Fang Tan, Xin Zhao

**Affiliations:** ^1^Chongqing Collaborative Innovation Center for Functional Food, Chongqing Engineering Research Center of Functional Food, Chongqing Engineering Laboratory for Research and Development of Functional Food, Chongqing University of Education, Chongqing 400067, China; ^2^School of Teacher Development, Chongqing University of Education, Chongqing 400067, China; ^3^Department of Pharmacy, Xindu District People's Hospital of Chengdu, Chengdu, 610500 Sichuan, China; ^4^School of Continuing Education, Chongqing University of Education, Chongqing 400067, China; ^5^Department of Education, Our Lady of Fatima University, Valenzuela 838, Philippines; ^6^Department of Public Health, Our Lady of Fatima University, 838 Valenzuela, Philippines

## Abstract

Yak yogurt is mainly produced in Qinghai-Tibet Plateau. It is a kind of naturally fermented dairy product. It contains abundant microorganisms. *Lactobacillus fermentum* (LF) HFY03 is a lactic acid bacteria derived from it. Our main research content is to study the influence of LF-HFY03 on the antifatigue and antioxidation ability of running exhausted mice. We gave different doses of LF-HFY03 to mice by gavage for 4 weeks. We selected vitamin C as the positive control group, mainly to study the relationship between antioxidant capacity and fatigue resistance and LF-HFY03 in mice with running exhaustion. The results showed that LF-HFY03 and vitamin C could significantly improve the running time of mice. And with the increase in LF-HFY03 concentration, the exhaustion time of mice was also extended. LF-HFY03 can reduce the content of urea nitrogen and lactic acid and also can increase the content of free fatty acids and liver glycogen. The levels of alanine aminotransferase, serum creatine kinase, and aspartate aminotransferase in mice decreased gradually as the antioxidant peptide level of walnut albumin increased. LF-HFY03 can reduce malondialdehyde (MDA) levels in a quantification-dependent manner and can also increase catalase (CAT) and superoxide dismutase (SOD) levels. LF-HFY03 can also increase the expressions of CAT mRNA, Cu/Zn-SOD, and Mn-SOD in the liver of mice. At the same time, LF-HFY03 can also increase the expression of protein of threonine transporter 1 (AST1)/alanine/cysteine/serine, mRNA, nNOS, and eNOS. At the same time, the solution could reduce the expression of TNF-*α*, syncytin-1, and inducible nitric oxide synthase (iNOS). The results showed that LF-HFY03 has a high development and application prospect as an antifatigue probiotic nutritional supplement.

## 1. Introduction

Due to its unique geographical environment, the Qinghai-Tibet Plateau has formed a dietary habitat, which is different from those in other regions. The yogurt produced by yak milk naturally fermented by local herdsmen contains rich microorganisms [1]. Lactic acid bacteria were found to be the main microorganism in yak yogurt fermented in Qinghai-Tibet Plateau, and most of them were new strains that were different from the known lactic acid bacteria [2]. They had strong resistance toward gastric acid and bile salt [3]. The study also showed that *Lactobacillus* in yak yogurt could reduce blood lipid concentration and serum cholesterol level and improve intestinal function, prevent hypertension and inflammation, regulate immune function, and inhibit oxidative stress [2–4]. It can be seen that yak yogurt has the potential to be used as microbial preparation and nutritional supplement, which needs to be further developed and utilized.

Exercise fatigue is a very common phenomenon for the urban fitness crowd, athletes, office workers, and students. Fatigue refers to a physiological phenomenon that occurs when the body is unable to maintain the intensity of exercise. The reasons for its formation are very complex, such as disease, exercise, mental factors, and irregular work and rest [5]. Exercise fatigue is usually closely related to the rate of material consumption and the high intensity of operation, which leads to the transformation of energy into many metabolites through metabolism, which are produced due to excessive accumulation [6]. In humans or animals, intense or overloaded exercise may cause cells to switch from aerobic respiration to anaerobic fermentation. In this state, the body will produce a large amount of lactic acid. Fatigue occurs when there is an imbalance in reactive oxygen species (ROS) levels, internal pH, and osmotic pressure [7]. According to the investigation results, ROS is the main factor that leads to the destruction of cell integrity, and then, oxidative skeletal muscle fatigue occurs with the influence of lipid peroxidation. At moderate concentrations, ROS play an important role as regulatory mediators in signaling processes [8].

It is found that lactic acid bacteria have a stronger biological activity and can be used as a probiotic [9]. According to some research data, the functions of lactic acid bacteria include adjuvant treatment of diseases, improvement of biological detoxification, and biological activity and immunity and can be used as nutritional supplements and microbial drug preparations [10, 11]. At the same time, lactic acid bacteria isolated from food are generally safer than synthetic drugs or hormone drugs [12]. Thus, they have a good application prospect among the elderly, athletes, patients, and other special groups [13]. However, the effect of lactic acid bacteria on exercise-induced fatigue is less studied, and its mechanism is still unclear. Therefore, this study observed the effects of LF-HFY03, which was isolated and identified by a research group on fatigue and antioxidant capacity of running exhausted mice so as to provide the necessary theoretical basis for the development of microbiological drugs and sports nutrition supplements.

## 2. Materials and Methods

### 2.1. Strain of LF-HFY03

LF-HFY03 is mainly produced in Qinghai-Tibet Plateau of China. It is a kind of lactic acid bacteria obtained by natural fermentation of yak such as yogurt and then by means of a separation process. It was identified as *Lactobacillus fermentum* by the 16S rRNA method and named as *Lactobacillus fermentum* HFY03 by our research group. The patented strains are mainly kept in China Microbial Culture General Preservation Center, Beijing, China (culture preservation number: CGMCC No. 16631).

### 2.2. Animal Grouping and Handling

The 75 Institute of Cancer Research (ICR) mice (six weeks old, male, 22 ± 2 g) were purchased from Chongqing Byrness Weil Biotech Ltd. (Chongqing, China, SCXK (XIANG) 2019-0004). Every five experiment mice were fed together in a cage, and the experiment mice were fed in a room at constant temperature (25°C ± 2°C) and humidity (50% ± 5%) with 12 h light and 12 h darkness, and the mice were given free access to standard mouse food and water. After one week of adaptive feeding, 75 mice were divided into five groups with 15 mice in each group. They were the high-dose LF-HFY03 group (LF-HFY03H), control group, low-dose LF-HFY03 (LF-HFY03L), running group, and vitamin C group, with 15 mice in each group. For the LF-HFY03 experimental group, different doses of LF-HFY03 were intragastric, namely, 1.0 × 10^8^ and 1.0 × 10^9^ CFU/kg. The vitamin C group was fed with 100 mg/kg vitamin C, while the running group and the control group were fed with 0.2 mL normal saline for 4 weeks.

### 2.3. Running Exhaustion Experiments

Mice in the LF-HFY03H group, the running group, the LF-HFY03L group, and the vitamin group were given treadmill training for half an hour every three days at a speed of 30 m/min from the point of intragastrium of LF-HFY03 and vitamin C. After the last intragastric administration of LF-HFY03, i.e., 4 weeks later, a fatigue test on mice was carried out. The mice ran on a treadmill until they were exhausted. The treadmill model is the ZH-PT animal experimental platform produced by Anhui Zhenghua Biological Instrument and Equipment Co., Ltd, China. The criterion for exhaustion was the inability of the mice to maintain a 30 m/min speed for more than three times on the run. Electricity, sound, light, and so on cannot play a stimulating role.

### 2.4. Energy Metabolism Index Measurement

The mouse liver was mixed with 0.5 mL perchloric acid and stirred well. However, centrifugation was performed (15°C, 15 min, 15000 r/min). The supernatant was removed and added to a 96-well plate with 30 *μ*L Glycogen Standard, adding 200 *μ*L KIODIDE to each well, which was left standing for 10 min. Liver glycogen was determined by setting the absorbance at 460 nm [14]. Serum lactic acid content (LAC) was determined mainly by the lactate oxidase method [15], serum urea nitrogen (BUN) content by diacetyl oxime colorimetry [16], and serum free fatty acid (NEFA) content by copper ion colorimetry [17].

### 2.5. Sports Injury Index Determination

After the exhaustive running experiment, mice were immediately killed by breaking the neck. Blood was collected from the heart. After centrifugation (4000 r/min, 20 min), the supernatant was taken. The levels of alanine aminotransferase, serum creatine, and aspartate aminotransferase were determined by the kit method (Solarbio Life Sciences, Beijing, China).

### 2.6. Antioxidant Index Determination

The activity levels of malondialdehyde (MDA), superoxide dismutase (SOD), and catalase (CAT) in serum of mice were determined by pyrogallol colorimetry, visible light method, and thiobarbituric acid colorimetry (Solarbio Life Sciences, Beijing, China) [18].

### 2.7. Hematoxylin and Eosin (H&E) Staining

Each group consisted of 15 mice, 10 of which underwent the exhaustive running test, and the remaining 5 did not undergo the exhaustive running test. After the mice were killed before and after the exhaustive running test, the liver was removed and rinsed with normal saline. Formalin solution at a concentration of 10% was fixed and embedded with paraffin. However, the sections were processed into 4 *μ*m thick sections and stained with hematoxylin and eosin (H&E) to evaluate the pathological changes of mouse livers. The staining was observed under a light microscope (BX43, Olympus, Tokyo, Japan).

### 2.8. qPCR Assay

The liver was removed and mixed with skeletal muscle, and TRIzol was used for RNA gamma. After extraction, the cDNA template was diluted to 1 *μ*L and obtained by the reverse transcription kit. 1 *μ*L forward and reverse primers (Table 1), 1 *μ*L cDNA template, 10 *μ*L SYBR Green PCR premix, and 7 *μ*L sterile distilled water were mixed uniformly, and the reaction time was 15 s at 95°C, 30 s at 55°C, 30 s at 95°C, and 35 s at 55°C. The relative gene expression was calculated with the help of the 2^-*ΔΔ*CT^ method, and the reference expression was glyceraldehyde dehydrogenase 3-phosphate (GAPDH) [19].

### 2.9. Western Blot

The mixture of 10 *μ*L PMSF, 1 mL radioimmunoprecipitation, and 100 mg bone tissue was stirred (12000 r/min) and then centrifugated (12000 r/min, 4°C, 15 min). The intermediate protein layer solution was removed, and the protein was quantified by the BCA protein quantification kit. The samples of each group were diluted to 50 *μ*g/mL, and the diluted protein was mixed with sample buffer at a mixing ratio of 4 : 1. The samples were heated at 100°C for 5 min and then treated in ice bath for 5 min. After completing the above steps, mix N,N,N′,N′-tetramethylenediamine (TEMED), acrylamide, 10% ammonium persulfate (APS), separation buffer, dissolved water, and Starking buffer in proportion. Concentrated gel (Thermo Fisher Scientific, Inc., Waltham, MA, USA) and N,N,N′,N′-tetramethylenediamine (TEMED) were mixed in proportion to prepare sodium dodecyl sulfate polyacrylamide gel electrophoresis (SDS-PAGE) separation gel. And it is imported into the rubber sheet to wait for use. The sample and the predyed Protein Ladder were introduced into the sample well, respectively, and then, vertical gel electrophoresis of SDS-PAGE adhesive was performed for 50 min. Polyvinylidene difluoroethylene (PVDF) was activated with methanol for 1 min and then transformed into a PVDF membrane. After conversion, a 1x TBST solution containing 5% skimmed milk was sealed for 1 hour. After sealing, it was cleaned and treated with 1x TBST and incubated for 2 h at 2°C. The PVDF membrane was clearly treated with 1x TBST, repeated five times, and incubated at 25°C for 1 h. After completing the above steps, the PVDF membrane is sprayed with super signal West Pico Plus and then placed into the imaging system for observation (iBright, Thermo Fisher Scientific) [20].

### 2.10. Statistical Analysis

All trials were performed three times, and the data were expressed as the mean ± standard deviation. At the same time, SPSS18.0 software is used to analyze the data. One-way analysis of variance (ANOVA) and least significant difference (LSD) tests were used as testing methods, and the significance level was *P* < 0.05.

## 3. Results

### 3.1. Running Exhaustion Time of Mice

**Figure 1:**
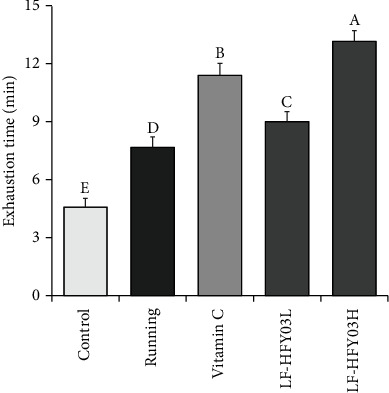
Comparison of exercise exhaustion time of mice in different groups. After passing the examination and analysis of (a–e), it was found that there were significant differences between the two groups with different superscripts (*P* < 0.05).

On analyzing the running exhaustion time of each group of mice (Figure 1), the different doses of LF-HFY03 solution and vitamin C were used for intragastric treatment of mice, lasting for 4 weeks. The results showed that the running exhaustion time of mice could be significantly improved, and the effect was significantly better than that of the running group and the control group. In addition, the elapsed time of the LF-HFY03H group was significantly higher than that of the vitamin C group, and with the increase of the concentration of the LF-HFY03 solution, the exhaustion time of mice also extended.

### 3.2. Energy Metabolism in Mice

The main energy metabolism indexes of mice in each group were measured after the trial run test.  Table 2 shows that the contents of free fatty acids and liver sugar in mice treated with different doses of LF-HFY03 and vitamin C were higher than those in the running group and the control group. At the same time, the contents of free fatty acids and glycogen in mice in the LF-HFY03H group were significantly higher than those in the vitamin C group, and the LF-HFY03 solution could improve the contents of free fatty acids and glycogen in mice in a quantification-dependent manner. However, compared with the running group, the contents of urea nitrogen and lactic acid in mice in different doses of the LF-HFY03 group and the vitamin C group were lower. With the increase of LF-HFY03 concentration, the contents of urea nitrogen and lactic acid in mice decreased gradually.

### 3.3. Sports Injury in Mice

In this study (Table 3), the results showed that the levels of ALT, CK, and AST in mice in different doses of the LF-HFY03 group and the vitamin C group were much higher than those in the running group. At the same time, the above three indexes of mice in the LF-HFY03H group were lower than those in the vitamin C group. At the same time, when the concentration of LF-HFY03 was continuously increased, the level of three-phase indexes in the mice would continuously decrease.

### 3.4. Serum Oxidation Level of Mice

As shown in Table 4, SOD and CAT levels in mice in the LF-HFY03 and vitamin C groups with different doses were significantly higher than those in the running group. The supplementation of LF-HFY03 can increase the CAT level and SOD level in mice in a quantification-dependent manner. However, the MDA level in mice in the LF-HFY03 and vitamin C groups with different doses was much higher than that in the running group, and the MDA level in mice began to decrease after the supplementation of LF-HFY03.

### 3.5. Pathological Observation

**Figure 2:**
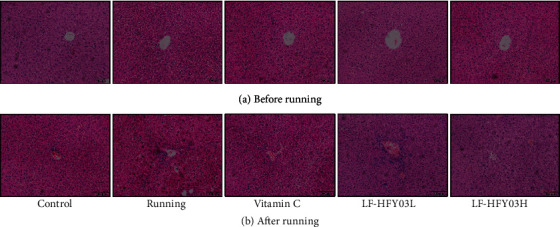
H&E slices of liver tissue in different groups before (a) and after (b) exercise.

Before the exhaustive running test, the study used H&E dyeing to evaluate the liver of mice. The results showed that (Figure 2(a)) the liver nucleus of each group of mice was evenly dyed. The cell structure of the liver tissue of mice was relatively normal, and the stem cells around the central vein showed a radiation distribution state, which indicated that the LF-HFY03 group and the vitamin C group did not produce very significant toxic and side effects on the liver of mice. After the exhaustive running test, the cells in each group (Figure 2(b)) were arranged unevenly to a certain extent, the central vein was also regular, and part of the cell structure was damaged and necrotized. Both vitamin C and LF-HFY03 can alleviate the liver damage caused by exhaustive running, and LF-HFY03H had the best effect.

### 3.6. mRNA Expression of Cu/Zn-SOD, Mn-SOD, and CAT in Mouse Liver

**Figure 3:**
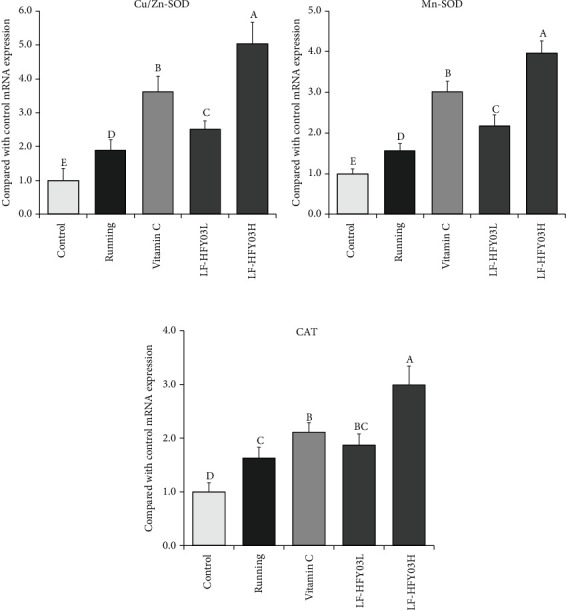
CAT, Cu/Zn-SOD, and Mn-SOD mRNA expression in mouse liver tissue. After passing the examination and analysis of (a–e), it was found that there were significant differences between the two groups with different superscripts (*P* < 0.05).

According to Figure 3, it can be found that the expressions of CAT mRNA, Cu/Zn-SOD, and Mn-SOD in the control group were the lowest, and the expressions of CAT mRNA, Cu/Zn-SOD, and Mn-SOD in the running group were higher than those in the control group. However, the expressions of CAT mRNA, Cu/Zn-SOD, and Mn-SOD in the LF-HFY03H group, the vitamin C group, and the LF-HFY03L and LF-HFY03L groups were higher than those in the running combination control group, and the highest expression was found in the LF-HFY03H group.

### 3.7. mRNA and Protein Expression of nNOS, eNOS, iNOS, Syncytin-1, ASCT1, and TNF-*α* in Mouse Skeletal Muscle

**Figure 4:**
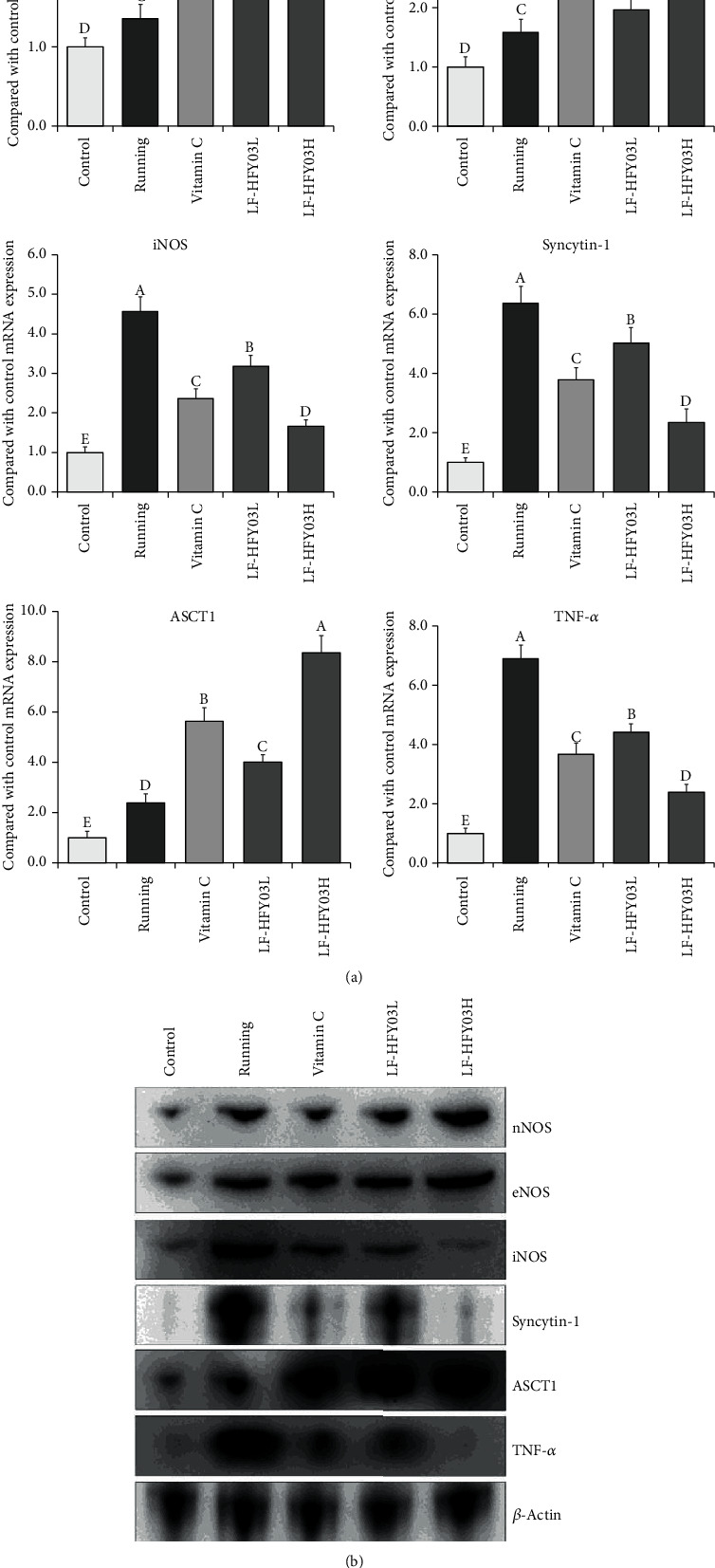
Expression of tissue protein, nNOS, eNOS, iNOS, syncytin-1, AST1, and TNF-*α* mRNA (a) and protein (b) in skeletal muscle of mice. After passing the examination and analysis of (A–E), it was found that there were significant differences between the two groups with different superscripts (*P* < 0.05).

In Figure 4, the expression intensity of tumor necrosis factor-alpha (TNF-*α*) mRNA, iNOS, and syncytin-1 and the protein in the skeletal muscle of each group of mice from high to low were set as follows: control group, LF-HFY03H group, running group, vitamin C group, and LF-HFY03L group. AST1, nNOS, and eNOS showed opposite trends. The expression intensity was set from high to low as follows: LF-HFY03H group, vitamin C group, LF-HFY03L group, running group, and control group.

## 4. Discussion

The mechanism of exercise-induced fatigue is complex, mainly including failure of the free radical attack, energy generation, and accumulation of metabolites [21]. The so-called energy exhaustion refers to the body because of excessive exercise and intense physical energy and material consumption, because of the lack of individual ability to supply and form a fatigue state. A way of energy supply is the accumulation of metabolites that can affect the body due to severe or excessive exercise, resulting in the conversion of aerobic energy supply into anaerobic digestion and the accumulation of a large number of anaerobic digestion metabolites such as lactate and ammonia, which damages the acid-base balance and causes an imbalance in the internal environment of the cell, thus leading to the formation of fatigue. According to the free radical attack, anaerobic digestion will lead to the increase of oxygen free radicals in the body, which will lead to the damage of cell membrane permeability and fluidity and also lead to tissue damage and fatigue [22].

Most of the pathological fatigue needs to be treated with drugs. However, in healthy people, exercise-induced fatigue is the most common. In general, the main side effects of supplements and treatments for people include physical therapy. Physical therapy mainly includes massage and rehabilitation exercise. However, physical therapy can only relieve fatigue in a short period of time, but it cannot improve the body's ability to resist fatigue. At present, the main research in the field of antifatigue focuses on nutritional supplements. Some research results have shown that some foods can play a role in antioxidant, immunity enhancement, and antiaging, as well as the prediction of cardiovascular disease. So, they can have a certain antifatigue effect [23]. However, no research has been reported on the effects of such foods on exercise-induced fatigue.

At present, *in vivo* animal tests are widely used to observe the antifatigue effect and the level of exercise ability of the target subject by means of detailed running tests [24]. This study mainly took mice as the research object and investigated and analyzed the antioxidant effect and antifatigue effect of LF-HFY03, while vitamin C was taken as the positive control group. At the same dose, the exhaustion time of LF-HFY03 was significantly higher than that of vitamin C. The depletion time of mice increased with the increase of LF-HFY03 concentration. It can be seen that LF-HFY03 can show a good antifatigue effect.

The supply of free fatty acids, liver glycogen, urea nitrogen, and lactic acid is correlated [25]. Liver glycogen is mainly stored in the liver; its main component is glucose molecules, after the breakdown of the body to provide the necessary energy. Therefore, the liver glycogen level is the key index to evaluate the fatigue degree of the body. Free fatty acids are the product of the breakdown of fats. If the body is in a state of long-term exercise or high-intensity exercise, the consumption of body fat will increase at this time, and the content of free fatty acids will increase to provide the energy needed by the body for exercise, thus achieving the purpose of relieving fatigue [26]. According to the results of the study, free fatty acid and liver glycogen levels were significantly higher in the mice after exhaustive exercise than in the running group and the control group. The level of acid in mice of the LF-HFY03H group was higher than that of the vitamin C group. In conclusion, the supplementation of LF-HFY03 can improve the content of free fatty acids and liver glycogen in the body in a dose-dependent manner. It is suggested that the supplementation of LF-HFY03 can improve the liver glycogen reserve capacity, promote fat mobilization, and alleviate fatigue.

After a long period of exercise or high-load exercise, the body will promote the anaerobic glycolysis process and then produce a large amount of lactic acid in the body. At this time, the acidity in the body will increase, leading to muscle contraction and other problems in the body. In addition, prolonged exercise and high-load exercise will also lead to protein degradation, resulting in the production of urea nitrogen, which is the main factor of protein consumption in the body [27, 28]. The results showed that after the exhaustive exercise experiment, the contents of urea nitrogen and lactic acid in mice in the LF-HFY03 group and the vitamin C group were much lower than those in the running group. However, the contents of urea nitrogen and lactic acid in mice in the LF-HFY03H group were lower than those in the vitamin C group. With the increase in the LF-HFY03 concentration, the contents of lactic acid and urea nitrogen decreased gradually. It can be concluded that supplementation of LF-HFY03 can effectively regulate energy metabolism, thus alleviating fatigue, reducing lactic acid accumulation, and achieving consistent protein degradation.

During exercise, the body's liver and muscles are vulnerable to damage. When the body is injured by excessive exercise, it is usually accompanied by an increase in ALT, CK, and AST levels [29]. According to the test results, the levels of ALT, serum CK, and AST in mice in the LF-HFY03 group and the vitamin C group were much higher than those in the running group. The levels of ALT, CK, and AST in the LF-HFY03H group were compared with those in the vitamin C group. With the increase in the LF-HFY03 concentration, the levels of the 3 indexes gradually decreased. It is suggested that LF-HFY03 has the function of preventing sports injury.

When the body moves excessively, superoxide anion free radicals will be produced inside the body, which can damage cell metabolism and cell membrane system, as well as produce body-like stress injury. CAT and SOD are key antioxidant enzymes in the body. In addition to having antioxidant effects, SOD also has the function of repairing damaged cells. In most vertebrates, the main expression forms of SOD are Mn-SOD and Cu/Zn-SOD [30]. In the body, SOD1 can convert superoxide radical (O_2_^-·^) into H_2_O_2_, which is relatively less toxic, and generate nontoxic H_2_O after CAT catalytic treatment, finally achieving the goal of eliminating free radicals [31–33]. An animal experiment also confirmed that there was a correlation between the enzyme contents of SOD1 and CAT, and the two contents were positively correlated [33]. In this study, the levels of CAT and SOD in mice in the LF-HFY03 group and the vitamin C group were much higher than those in the running group, and a certain dose of LF-HFY03 supplement could significantly improve the levels of CAT and SOD in mice. Therefore, it can be shown that LF-HFY03 has antioxidant capacity. As a product of cell membrane lipids after sampling, MDA can be regarded as an important index parameter after lipid peroxidation. If the body is in a state of fatigue, the MDA level in the body will be significantly increased [34]. In this study, the MDA level in mice in the LF-HFY03 group and the vitamin C group was much lower than that in the running group, and a certain dose of LF-HFY03 supplement could effectively reduce the MDA level in mice. In conclusion, LF-HFY03 can reduce lipid peroxidation and then play the role of antioxidant and cell protection.

nNOS protects tissues and nerve cells and can repair tissue damage caused by oxidative damage [35]. The expression of eNOS was relatively stable in tissues, and eNOS produces NO, which promotes the repair of liver damage caused by oxidative stress [36]. When iNOS is activated, the enzyme activity can be maintained for a long time, accompanied by the release of a large amount of NO. Excessive levels of NO can lead to genetic mutations and tissue damage [37]. Oxidative stress aggravates inflammation, resulting in overexpression of iNOS, insufficient expression of nNOS and eNOS, and aggravation of tissue damage [38]. Studies have shown that the expression of syncytin-1 will be significant when muscle tissue is damaged, inflamed, and atrophic, thus affecting the exercise ability [39]. The increased expression of syncytin-1 resulted in the inhibition of ASCT1 expression, and NO can regulate the expression of AST1 [40]. When the body has an inflammatory reaction, the iNOS level is increased, resulting in a large number of NO production, thus inhibiting the expression of ASCT1 [39, 41]. However, TNF-*α* induces and enhances the expression of syncytin-1 in muscle tissue, leading to the production of inflammatory cytokines and free radicals and thereby inhibiting the expression of ASCT1 [42]. In this study, both LF-HFY03 and vitamin C can control the expression of AST1 in skeletal muscle of mice after intense exercise and meanwhile reduce the expression of TNF-*α*, syncytin-1, and iNOS. The same results were obtained with previous studies, suggesting that the LF-HFY03 is designed to alleviate muscle tissue damage caused by exhaustive exercise.

Research shows that high-quality animal milk is a strong antioxidant [43]. The yogurt fermented by animal milk under the action of lactic acid bacteria not only improves its antioxidant activity but also better retains the strains with excellent antioxidant activity due to the domestication of lactic acid bacteria in the process of long-term natural fermentation [44]. Lactic acid can adjust the composition of intestinal flora, thus improving the body's immunity and promoting digestion and absorption [45], as lactic acid bacteria can improve human health in many ways. According to many scientific verifications, it has been found that some lactic acid bacteria can play a role in alleviating fatigue, but there are relatively few studies on the mechanism of action [46]. In addition, according to some research results, lactic acid bacteria have antioxidant functions in both internal and external links of the body [47, 48]. This study also confirmed that LF-HFY03 has a good antioxidant effect and an antifatigue effect *in vivo*. In this study, some mechanisms have been explained, and more in-depth research is needed in the future.

## 5. Conclusions

It can be concluded that LF-HFY03 has a good antifatigue and antioxidation ability, and its antifatigue properties are associated with reduced protein breakdown, improved liver glycogen storage capacity, reduced lactic acid accumulation, and increased fat consumption. Its antioxidant capacity is closely associated with reducing lipid peroxidation and scavenging free radicals. LF-HFY03 has a high development and application prospect as a sports nutrition supplement or pharmaceutical microbial preparation. This study has accumulated a theoretical basis for its application under such aspects.

## Figures and Tables

**Table 1:** Sequences of the primers used for this experiment.

Gene name	Sequence
*Cu/Zn-SOD*	Forward: 5′-AACCAGTTGTGTTGTCAGGAC-3′
	Reverse: 5′-CCACCATGTTTCTTAGAGTGAGG-3′
*Mn-SOD*	Forward: 5′-CAGACCTGCCTTACGACTATGG-3′
	Reverse: 5′-CTCGGTGGCGTTGAGATTGTT-3′
*CAT*	Forward: 5′-GGAGGCGGGAACCCAATAG-3′
	Reverse: 5′-GTGTGCCATCTCGTCAGTGAA-3′
*nNOS*	Forward: 5′-TCGTCCAACTTCTGGGCTCTT-3′
	Reverse: 5′-CCTTCTCTTCCTCCCCTCTCTTC-3′
*eNOS*	Forward: 5′-TCAGCCATCACAGTGTTCCC-3′
	Reverse: 5′-ATAGCCCGCATAGCGTATCAG-3′
*iNOS*	Forward: 5′-CAAAGGCTGTGAGTCCTGCAC-3′
	Reverse: 5′-ACTTTGATCAGAAGCTGTCCC-3′
*Syncytin-1*	Forward: 5′-GTTAACTTTGTCTCTTCCAGAATCGA-3′
	Reverse: 5′-CATCAGTACGTGGGCTAGCA-3′
*ASCT1*	Forward: 5′-ACGCGGGACAGATTTTCAC-3′
	Reverse: 5′-ACACCCGCTGCTCCAAC-3′
*TNF-α*	Forward: 5′-TGCCACTTCATACCAGGAGA-3′
	Reverse: 5′-CCGGAGTCCGTGATGTCTA-3′
*GAPDH*	Forward: 5′-GAAGGTGAAGGTCGGAGTCA-3′
	Reverse: 5′-AATGAAGGGGTCATTGATGG-3′

**Table 2:** Hepatic glycogen, lactic acid, blood urea nitrogen, and free fatty acid levels of mouse in each group.

Group	Hepatic glycogen (mg/g)	Lactic acid (mg/L)	Blood urea nitrogen (mg/L)	Free fatty acid (*μ*mol/mL)
Control	3.25 ± 0.52^e^	0.29 ± 0.06^e^	115.35 ± 6.89^e^	339.92 ± 18.63^e^
Running	5.15 ± 0.61^d^	1.17 ± 0.16^a^	336.90 ± 11.52^a^	1658.74 ± 87.20^a^
Vitamin C	9.23 ± 0.69^b^	0.63 ± 0.06^c^	205.63 ± 8.95^c^	945.69 ± 66.90^c^
LF-HFY03L	7.70 ± 0.52^c^	0.82 ± 0.08^b^	264.14 ± 9.03^b^	1255.03 ± 72.06^b^
LF-HFY03H	15.06 ± 0.58^a^	0.43 ± 0.06^d^	167.92 ± 6.96^d^	569.35 ± 36.97^d^

“±” for standard deviation. ^a-e^After Tukey's honestly significant difference test analysis, there is a significant difference between the two groups with different superscripts (*P* < 0.05).

**Table 3:** Serum CK, AST, and ALT levels of mouse in each group.

Group	CK (U/L)	AST (U/L)	ALT (U/L)
Control	95.20 ± 5.03^e^	14.63 ± 1.15^e^	8.93 ± 1.21^e^
Running	356.72 ± 21.08^a^	108.36 ± 6.30^a^	89.63 ± 4.36^a^
Vitamin C	210.36 ± 15.39^c^	52.93 ± 4.69^c^	44.50 ± 3.87^c^
LF-HFY03L	286.02 ± 18.33^b^	77.03 ± 5.23^b^	65.05 ± 4.11^b^
LF-HFY03H	155.92 ± 10.30^d^	30.82 ± 2.99^d^	18.93 ± 1.88^d^

“±” for standard deviation. ^a–e^After Tukey's honestly significant difference test analysis, there is a significant difference between the two groups with different superscripts (*P* < 0.05).

**Table 4:** Serum SOD, CAT, and MDA levels of mice in each group.

Group	SOD (U/L)	CAT (U/L)	MDA (*μ*mol/L)
Control	40.89 ± 3.69^e^	28.36 ± 1.91^e^	2.88 ± 0.42^e^
Running	61.02 ± 4.08^d^	44.28 ± 2.97^d^	16.98 ± 2.26^a^
Vitamin C	125.68 ± 11.08^b^	108.36 ± 4.90^b^	6.82 ± 0.70^c^
LF-HFY03L	86.36 ± 7.03^c^	74.52 ± 5.88^c^	11.36 ± 1.02^b^
LF-HFY03H	172.08 ± 8.69^a^	145.25 ± 10.02^a^	4.03 ± 0.32^d^

“±” for standard deviation. ^a–e^After Tukey's honestly significant difference test analysis, there is a significant difference between the two groups with different superscripts (*P* < 0.05).

## Data Availability

The datasets generated for this study are available upon request to the corresponding authors.
